# Evaluation of the protective effect of N-acetylcysteine on contrast media nephropathy

**DOI:** 10.12861/jrip.2015.23

**Published:** 2015-09-01

**Authors:** Aiyoub Pezeshgi, Negin Parsamanesh, Goodarz Farhood, Khalil Mahmoodi

**Affiliations:** ^1^Zanjan Metabolic Disease Research Center, Zanjan University of Medical Sciences, Zanjan, Iran; ^2^Department of Internal Medicine, School of Medicine, Zanjan University of Medical Sciences, Zanjan, Iran

**Keywords:** Contrast induced nephropathy, Acute kidney injury, Contrast agents

## Abstract

**Introduction:** Intravenous contrast agents can cause acute decline in kidney function, especially in patients with risk factors.

**Objectives:** In this study, we aimed to examine the ameliorative effect N-acetylcysteine (NAC) to reduce the incidence of contrast nephropathy.

**Patients and Methods:** This study was a prospective, randomized, double-blind clinical trial on 150 patients who underwent coronary angiography. The study was carried out on patients undergoing coronary angiography. Patients were randomly assigned into 2 groups of intervention group and control subjects. Intervention group took NAC 600 mg orally twice a day. It was administered one day before angiography and continued until the second day after angiography. Control subjects received saline only. Serum creatinine was measured before and three days after coronary angiography.

**Results:** There was no significant difference between intervention and control groups at baseline (*P* > 0.05). However, there was a significant decline in creatinine level among NAC patients (*P* = 0.001). Saline group had significantly higher proportion of nephropathy cases than NAC patients

**Conclusion:** We found that the consumption of NAC is useful for contrast induced nephropathy (CIN) prevention.

**Implication for health policy/practice/research/medical education:**

In a prospective, randomized, double-blind clinical trial on 150 patients who underwent coronary angiography, we found that the use of N-acetylcysteine (NAC) is useful for contrast induced nephropathy (CIN) prevention.

## Introduction

Intravenous contrast agents can cause the acute decline in kidney function, especially in patients with risk factors (1). Contrast induced nephropathy (CIN) is the third most common cause of inpatient acute kidney injury (AKI) (2-4). CIN occurs 2 to 3 days after intravascular administration of iodinated contrast material. CIN is defined as the presence of 0.5 mg/dl creatinine or more than 25% rise in baseline creatinine during 48 hours after receiving the contrast agent (3,4). Serum creatinine usually reach to peak on the third to fifth day of administration and returns to the initial value on the seventh day. It is asymptomatic and non-oliguric, except in some cases in which the peak of creatinine is observed between fifth and tenth days, which returns to baseline on the second to third weeks (6). In rare cases of CIN, patients may require replacement therapy. CIN increases mortality and morbidity rate in the first year, especially when the patient needs replacement therapy (7). It have always been assumed that the risk of CIN can be reduced via using preventive methods such as full hydration by intravenous saline or oral fluid intake, sodium bicarbonate, vasodilators, various diuretics (furosemide or mannitol), calcium channel blockers, dopamine, theophylline, N-acetylcysteine (NAC), vitamins E and C, and also hemodiafiltration (8-15). Most patients who undergo coronary angiography are elderly patients with risk factors such as diabetes, hypertension, congestive heart failure, and chronic kidney disease, which predispose them to CIN. In most cases, appropriate hydration is not performed to deal with the underlying disease of such patients (16).

## Objectives

In this study, we aimed to examine the ameliorative effect of NAC to reduce the incidence of contrast nephropathy.

## Patients and Methods

Our study was a prospective, randomized, double-blind clinical trial. The study was carried out on patients undergoing coronary angiography in Ayatollah-Mousavi hospital, Zanjan, in 2012. Patients were randomly assigned into 2 groups of NAC and control subjects. They were randomly divided into 2 groups The control group received normal saline only and the case group received normal saline and oral NAC 600 mg twice daily.

Information on age, gender, history of diseases and medications were recorded. Both groups were hydrated with saline. Case group took NAC 600 mg orally twice a day. It was administered one day before angiography and continued until the second day after angiography. Serum creatinine was measured before and three days after coronary angiography. Researchers and patients were blind to patients’ groups. All patients received a unique brand and a fixed dose of low-osmolar contrast media. The patients continued to take their previously used medications. During the first three days we excluded patients who had a change in their medications or their mean arterial blood pressure when became less than 85 mm Hg.

### Ethical issues

The research followed the tenets of the Declaration of Helsinki. Informed consent was obtained. The research was approved by the Ethics Committee of Zanjan Medical University.

### Data analysis

Statistical analysis was performed using SPSS version 15 (SPSS Inc., Chicago, IL) software package. Descriptive statistics were used to summarize the data. Fisher exact test, chi-square test, *t* test and analysis of variance (ANOVA) were used. A *P *< 0.05 was considered significant.

## Results

Out of a total of 150 patients who were enrolled in the study, 83 patients were female and 67 patients were male. They was 75 peoples in each group. Table 1 shows mean creatinine level based on related variables in intervention and control groups at baseline.

**Table 1:** Mean serum creatinine level by study group at baseline

**Variable**		**Group**			*** P *** value
		**Saline normal** **n = 75** **Mean ± SD**	**NAC** **n = 75** **Mean ± SD**	**Total** **n = 150** **Mean ± SD**	
Gender	Female (n = 83)	1.17±0.40	1.17±0.39	1.17±0.39	0.064
	Male (n = 67)	1.25±0.26	1.31±0.31	1.28±0.39	
Hypertension	More than 140 mm Hg (n = 107)	1.29±0.38	1.22±0.35	1.25±0.36	0.074
	Lower than 139 (n = 43)	1.05±0.20	1.27±0.42	1.13±0.31	
Diabetes mellitus	Yes (n = 109)	1.21±0.35	1.17±0.35	1.19±0.35	0.151
	No (n = 41)	1.20±0.31	1.33±0.37	1.28±0.35	
ARB receivers	Yes (n = 15)	1.37±0.18	1.28±0.39	1.30±0.35	0.32
	No (n = 135)	1.20±0.35	1.22±0.36	1.21±0.35	
ACE receivers	Yes (n = 79)	1.19±0.25	1.22±0.34	1.20±0.29	0.613
	No (n = 71)	1.23±0.48	1.23±0.38	1.23±0.49	
	Mean	1.20±0.34	1.23±0.36	‏-	0.71

There was no significant difference of serum creatinine between intervention and control groups at base line (*P* > 0.05; Table 1).

Mean creatinine level at the end of the study in intervention and control groups is shown in Table 2 . For either the 2 groups, males experienced a higher reduction of creatinine level. Regardless of the history of hypertension, diabetes, angiotensin II receptor blockers (ARBs), or angiotensin-converting enzyme (ACE) inhibitor drugs, the mean level of creatinine was declined in the intervention group than control group, though the difference was not significant. However, there was a significant decline in creatinine level from base among NAC patients (*P *= 0.001; Table 2 ).

**Table 2:** Mean serum creatinine level by study group at the end of intervention

**Variable**		**Group**			***P*** ** value **
		**Saline normal** **n=75** **Mean ± SD**	**NAC** **n=75** **Mean ± SD**	**total** **n=150** **Mean ± SD**	
Gender	Female (n = 83)	1.30±0.50	1.07±0.38	1.18±0.45	0.14
	Male (n = 67)	1.36±0.30	1.17±0.31	1.27±0.32	
Hypertension	More than 140 mm Hg (n = 107)	1.42±0.47	1.10±0.33	1.24±0.43	0.27
	Lower than 139 (n = 43)	1.17±0.21	1.14±0.44	1.16±0.31	
Diabetes mellitus	Yes (n = 109)	1.34±0.44	1.08±0.34	1.22±0.42	0.91
	No (n = 41)	1.30±0.30	1.17±0.36	1.21±0.35	
ARB receivers	Yes (n = 15)	1.35±0.12	1.12±0.38	1.18±0.34	0.71
	No (n = 135)	1.33±0.42	1.11±0.35	1.22±0.40	
ACE receivers	Yes (n = 79)	1.31±0.27	1.14±0.35	1.25±0.31	0.33
	No (n = 71)	1.36±0.62	1.09±0.36	1.19±0.48	
	Mean	1.33±0.41	1.11±0.35	‏-	0.001

Table 3 shows the frequency of nephropathy cases among the studied groups who had a history of taking ARB or ACE drugs. Neither NAC nor saline group who had a history of administration of ARB showed signs of nephropathy (*P* > 0.5). However, there was a significant higher proportion of nephropathy cases in saline group (*P *= 0.001;  Table 3).

**Table 3:** Comparison of nephropathy frequency in the studied groups by ACE and ARB prescription

**Group**			**Nephropathy**		***P*** ** value**
			Yes n = 14	No n = 136	
Normal saline receivers	ACE	Yes (n = 50)	8	42	0.44
		No (n = 25)	5	20	
	ARB	Yes (n = 4)	0	4	0.45
		No (n = 71)	13	58	
NAC receivers	ACE receivers	Yes (n = 29)	0	29	0.61
		No (n = 46)	1	45	
	ARB receivers	Yes (n = 11)	0	11	0.85
		No (n = 64)	1	63	
Total		Saline normal (n = 75)	13	62	0.001
		NAC receivers (n = 75)	1	74	

Table 4 shows univariate analysis of variance of some variables and their main effects on serum creatinine level at the end of study. As shown, NAC participants experienced significantly higher decline in creatinine level (*P *= 0.001; Table 4).

**Table 4:** Analysis of the variables associated with serum creatinine level at the end of study

**Variable**	***P*** ** value**
Gender (Female versus Male)	0.29
Hypertension	0.13
Diabetes mellitus	0.85
ARB receivers	0.99
ACE receivers	0.90
Group (NAC versus normal saline)	0.001

## Discussion

Several clinical trial and meta-analysis have approved or rejected the effect of NAC on intravenous contrast material-induced nephropathy. In 2004 Bagshaw et al (9), published a meta-analysis, which covered 1261 patients in 14 studies. Only in 5 studies the incidence of CIN was lower after administration of NAC. Other studies showed no effect. In general, they did not express any related finding (9). In 2005 van den Berk et al (17), published a meta-analysis in Amsterdam, Netherlands. In their study, 5 out of the 16 studies showed the significant effects of NAC. In another study, the protective effect of NAC with a dose of 1200 mg twice daily was more than that with a dose of 600 mg (18). In 2004, Liu et al conducted a meta-analysis study at the University of California from 1974 to 2004, which included 9 randomized controlled trials (RCTs). He concluded that NAC was effective for the prevention of CIN because it was low- risk and low cost, and was advisable to use (19). Likewise, in 2007 Gonzales et al (20), conducted a meta-analysis which included 22 studies with 2746 patients, however the results did not support our idea. Acetylcysteine for contrast-induced nephropathy Trial (ACT), is the largest RCT. During 2008 and 2009 a study was conducted in 35 centers in Brazil, and 2300 high-risk patients were evaluated for CIN and underwent coronary angiography. The patients who received 1200 mg of oral NAC were compared with the placebo group. Of all, 13% of patients had heart failure and 18% had the renal failure (serum creatinine more than 1.5 mg/dl). It showed the reduction of the incidence of CIN (10). Additionally in a meta-analysis by Kwok et al (12), which included seven and nine RCT systematic reviews (15976 patients), a significant reduction of CIN risk was reported by administration of NAC, which supported its protective effect. In our study, after oral administration of NAC, mean serum creatinine did not increase three days after receiving the contrast, and rather it decreased. The frequency of contrast nephropathy in patients receiving NAC is lower. Thus the protective effect was achieved.

## Conclusion

We found that the administration of NAC is useful for CIN prevention.

## Limitations of the study

We lost a number of patients because they refused to continue the study or did not refer three days after taking contrast media. In addition, many of the patients were living in far distances, and we did not plan to take samples at their location. Hence, our conclusion was limited to a small number of patients.

## Authors’ contribution

AP and NP conducted the research and prepared the primary draft. GF and KM revised the manuscript. AP further edited the paper. All authors read and signed the paper.

## Conflicts of Interest

The authors declared no competing interests.

## Ethical considerations

Ethical issues (including plagiarism, misconduct, data fabrication, falsification, double publication or submission, redundancy) have been completely observed by the authors.

## Funding/support

This study was supported by a grant from Zanjan Medical University (Grant No. 19/3-3/2377).
